# A systematic review and meta-analysis investigating the relationship between metabolic syndrome and the incidence of thyroid diseases

**DOI:** 10.1007/s12020-023-03503-7

**Published:** 2023-09-09

**Authors:** Heba Alwan, Valerie Aponte Ribero, Orestis Efthimiou, Cinzia Del Giovane, Nicolas Rodondi, Leonidas Duntas

**Affiliations:** 1 Institute of Primary Health Care (BIHAM), University of Bern, Bern, Switzerland; 2 Graduate School for Health Sciences, University of Bern, Mittelstrasse 43, 3012 Bern, Switzerland; 3 Institute of Social and Preventive Medicine (ISPM), University of Bern, Bern, Switzerland; 4 Department of General Internal Medicine, Inselspital, Bern University Hospital, University of Bern, Bern, Switzerland; 5 Thyroid Section, Unit of Endocrinology, Diabetes and Metabolism, Evgenideion Hospital, National and Kapodistrian University of Athens, PC 11528 Athens, Greece

**Keywords:** Metabolic syndrome, Thyroid disorders, Diabetes, Incidence

## Abstract

**Purpose:**

To assess the prospective association between metabolic syndrome (MetS), its components, and incidence of thyroid disorders by conducting a systematic review and meta-analysis.

**Methods:**

A systematic search was performed in Ovid Medline, Embase.com, and Cochrane CENTRAL from inception to February 22, 2023. Publications from prospective studies were included if they provided data on baseline MetS status or one of its components and assessed the incidence of thyroid disorders over time. A random effects meta-analysis was conducted to calculate the odds ratio (OR) for developing thyroid disorders.

**Results:**

After full-text screening of 2927 articles, seven studies met our inclusion criteria. Two of these studies assessed MetS as an exposure (*N* = 71,727) and were included in our meta-analysis. The association between MetS at baseline and incidence of overt hypothyroidism at follow-up yielded an OR of 0.78 (95% confidence interval [CI]: 0.52–1.16 for two studies, I^2^ = 0%). Pooled analysis was not possible for subclinical hypothyroidism, due to large heterogeneity (I^2^ = 92.3%), nor for hyperthyroidism, as only one study assessed this association. We found evidence of an increased risk of overt (RR: 3.10 (1.56–4.64, I^2^ = 0%) and subclinical hypothyroidism (RR 1.50 (1.05–1.94), I^2^ = 0%) in individuals with obesity at baseline. There was a lower odds of developing overt hyperthyroidism in individuals with prediabetes at baseline (OR: 0.68 (0.47–0.98), I^2^ = 0%).

**Conclusions:**

We were unable to draw firm conclusions regarding the association between MetS and the incidence of thyroid disorders due to the limited number of available studies and the presence of important heterogeneity in reporting results. However, we did find an association between obesity at baseline and incidence of overt and subclinical hypothyroidism.

## Introduction

Non-communicable diseases (NCDs) are the leading cause of disease burden worldwide [1]. In 2022, NCDs accounted for more than 41 million deaths each year [2], thus killing more people than all other causes of mortality combined [1]. Other than their health impacts, NCDs also have both economic and social implications [1]. More than 80% of deaths from NCDs are due to cardiovascular diseases (CVDs), diabetes, cancer, and chronic respiratory diseases, which in turn tend to share common risk factors such as high blood pressure, tobacco use, unhealthy diet, physical inactivity, and the harmful use of alcohol [1]. Metabolic syndrome (MetS) is a cluster of factors that together increase an individual’s risk of developing CVDs and diabetes [3, 4]. The prevalence of MetS is increasing and it is currently estimated that around one quarter of the world’s population has MetS [5, 6].

Apart from an increased risk of CVDs and diabetes in individuals with MetS, thyroid disorders and MetS tend to co-exist, particularly among older adults [7]. Thyroid diseases are frequently encountered in clinical practice affecting up to 10% of older adults [8] and, in turn, have been associated with several adverse health events including increased risk of CVD, both in their overt and subclinical forms [7, 9]. The majority of the literature centered around the association between MetS and thyroid diseases is based on cross-sectional studies that have assessed the prevalence of MetS in individuals with thyroid disease [10–13]. These studies have demonstrated a positive association between these two conditions [10–13]. Cross-sectional studies have additionally revealed an association between hypothyroidism (or subclinical hypothyroidism) and components of MetS including diabetes [14], obesity [15], high blood pressure [16], and cholesterol levels [17]. However, the temporal relationship between thyroid disorders and MetS remains unclear [18]. Evidence from longitudinal studies in this field is relatively scarce, and most studies have investigated whether thyroid disorders are prospectively associated with the development of MetS or its components [19–22]. Interestingly, one study reported evidence of an association between subclinical hypothyroidism and prevalent, but not incident, MetS [20]. More recently, it has been suggested that the association might be bidirectional, or that it points in the opposite direction; i.e. that MetS, or insulin resistance, may be causally associated with the development of thyroid diseases [23, 24]. Few prospective studies have assessed the association of MetS at baseline and incidence of thyroid diseases and results were inconclusive [18, 25]. One Chinese study conducted among 66,822 participants found that individuals with MetS were more likely to develop subclinical hypothyroidism during a mean follow-up of 4.2 years (adjusted hazard ratio 1.21, 95% confidence interval [CI]: 1.03–1.42) [18]. Conversely, a cohort study performed in Iran among 4905 participants found no evidence of an association between MetS at baseline and incidence of overt or subclinical thyroid dysfunction (incidence rate ratio for overt hypothyroidism 0.72 (0.43, 1.15)) [25].

In the present study, we aimed to perform a systematic review and meta-analysis of the literature to investigate the prospective association between MetS, and its components, and incidence of thyroid disorders.

## Methods

The study protocol for this systematic review and meta-analysis was registered in the international Prospective Register of Systematic Reviews PROSPERO (CRD 42023407674). We adhered to the Preferred Reporting Items for Systematic reviews and Meta-Analyses (PRISMA) statement [26].

### Search strategy and selection criteria

We performed a systematic literature search in Ovid Medline, Embase.com, and in the Cochrane Central Register of Controlled Trials (CENTRAL) from inception to February 22, 2023. Prospective studies were included if they had data on baseline MetS status (or one of its components) and assessed the incidence of thyroid disorders at follow-up. The search strategy included terms related to the exposure (e.g., MetS, abdominal obesity, hypertension, hyperglycemia, and dyslipidemia) and the outcome (e.g., thyroid disease, thyroid function, hyperthyroidism, and hypothyroidism). The full search strategies for all databases are provided in the Appendix. We excluded studies that: (i) did not have a control group, (ii) had no data on thyroid disease at baseline, (iii) included persons less than 18 years of age or pregnant women, and (iv) only included individuals with type 1 diabetes. No language or time restrictions were applied. Reference lists of included studies were assessed for additional relevant articles. Two authors (H.A. and V.A.R.) independently screened all references for eligibility and discrepancies were resolved by discussion and consensus.

### Data extraction and quality assessment

Data were extracted by one author (H.A.). For each study, the following data were extracted: first author’s name, publication year, study country, study design, age, sample size, follow-up time, type of exposure, type of outcome, comparison group, and crude number of events or (adjusted or unadjusted) effect estimate for the relevant outcome. Risk of bias assessment was independently performed by two authors (H.A. and V.A.R.) using the Newcastle-Ottawa Scale (NOS) [27]. The NOS contains eight items divided into three categories: Selection, Comparability, and Outcome. Studies were given a score that ranged from 0 to 9 stars. A higher score indicated better methodological quality. Studies were thereafter classified into good, fair, and poor quality according to their star rating.

### Exposures

A number of diagnostic criteria proposed by various expert groups have been developed to define MetS [5, 28]. However, most of these criteria require the following conditions to coexist to fulfill the definition of MetS: abdominal obesity, insulin resistance, high blood pressure, low serum high-density cholesterol (HDL-C) levels, and elevated levels of serum triglycerides [5]. For the current analysis, the presence of MetS was determined according to the definition used by study authors. We also assessed, as exposures, individual components of MetS as defined by the authors of the analyzed studies; these included diabetes, prediabetes, hypertension, dyslipidemia, and abdominal obesity (or if not available, obesity defined as a body mass index of ≥30 kg/m^2^).

### Outcomes

The four possible outcomes that we investigated were overt hypothyroidism, subclinical hypothyroidism, overt hyperthyroidism, or subclinical hyperthyroidism. Thyroid-stimulating hormone (TSH) and free thyroxine (FT4) level cut-offs to define the four categories of thyroid status at follow-up were determined by each respective study. Studies that reported combined results for overt and subclinical hypo/hyperthyroidism were not included in the present analysis.

### Statistical analysis

We aimed to meta-analyze adjusted odds ratios (ORs), relative risks (RR) or hazard ratios (HR) separately, using a random effects model. We planned to pool ORs with RRs only if the outcome was considered a rare event. When it was not possible to pool adjusted effect estimates, we calculated unadjusted ORs from raw outcome data.

Heterogeneity was estimated using I^2^, tau, and the Q test. A post-hoc decision was made to refrain from pooling results together when considerable heterogeneity was present (I^2^ > 70%). We planned to explore publication bias via funnel plots and Egger’s test, if 10 or more studies were identified in a meta-analysis. All analyses were conducted using Stata 16.0 (StataCorp LP, College Station, TX, USA).

## Results

The literature search identified 2927 potentially relevant citations, of which 17 articles met our inclusion criteria (Supplementary Fig. 1) after title and abstract screening. Following the full-text screening, we identified eight articles that could be included in the qualitative synthesis [18, 25, 29–34] and seven articles that could be included in the quantitative synthesis [18, 25, 29–33]. Characteristics of the seven studies included in the meta-analysis are shown in Table 1. We were able to pool ORs for the association between MetS and incidence of overt and subclinical hypothyroidism, for the association between diabetes mellitus and prediabetes and overt and subclinical hypo/hyperthyroidism, and for the association between obesity and overt and subclinical hypothyroidism. We decided post-hoc to refrain from analyzing the association between some components of the MetS (hypertension, and dyslipidemia) and thyroid disorders due to the limited number of studies and/or extreme heterogeneity (details below).

**Table 1:** Characteristics of the studies included in the quantitative analysis

Author	Year	Country	Mean age	Total number of participants	Exposure	Outcome	Mean follow-up time (years)	Adjustment factors
Amouzegar [29]	2017	Iran	40.4	5783	Obesity	Ohypo, Shypo	6.3*	Not known
Chang [18]	2017	China	41.2	66,822	MetS, components	Ohypo, Shypo	4.2	No adjustment - crude data used
Chang [30]	2017	China	41.2	72,003	Diabetes, prediabetes	Ohypo, Shypo, Ohyper, Shyper	2.6*	No adjustment - crude data used
Dehaki [33]	2017	Iran	≥30**	1710	Diabetes, prediabetes	Ohypo, Shypo, Ohyper, Shyper	9	Age, sex, smoking, blood pressure, body mass index, thyroid peroxidase antibody, insulin resistance index, triglycerides, and cholesterol
Gopinath [32]	2008	Australia	68.8	1063	Diabetes	Ohypo, Shypo, Ohyper, Shyper	10	No adjustment - crude data used
Gopinath [31]	2010	Australia	67.6	951	Obesity	Ohypo, Shypo	5	Age, sex
Mehran [25]	2020	Iran	40.4	4905	MetS	Ohypo, Shypo, Ohyper, Shyper	9.7	No adjustment - crude data used

^*^Median; **Mean age of total study population not reported

*MetS* Metabolic syndrome, *Ohypo* Overt hypothyroidism, *Shypo* Subclinical hypothyroidism, *Ohyper* Overt hyperthyroidism, *Shyper* Subclinical hyperthyroidism

### Quality assessment

The quality of all but two studies was deemed to be poor according to the NOS (Supplementary Table 1). This was mainly because most effect estimates used in our analyses were unadjusted, in order to make it possible to pool results from the included studies. We were unable to assess publication bias due to the small number of studies included in the meta-analyses.

### MetS

**Fig. 1:**
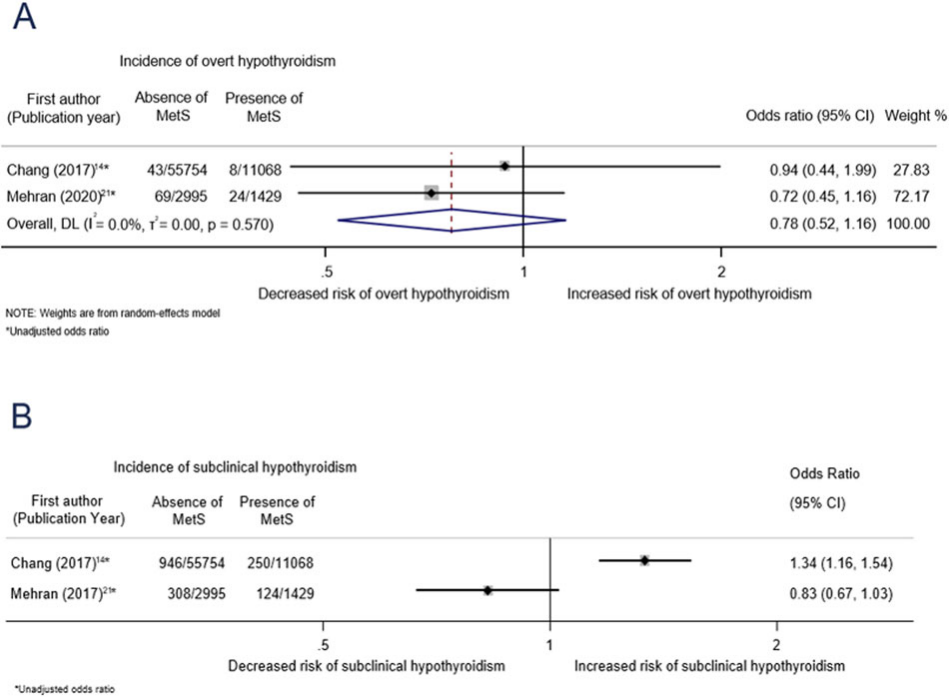
Association between metabolic syndrome at baseline and hypothyroidism at follow-up. **A** Association between metabolic syndrome and incidence of overt hypothyroidism. **B** Association between metabolic syndrome and incidence of subclinical hypothyroidism. MetS metabolic syndrome

The pooled unadjusted OR for the association between MetS and incidence of overt hypothyroidism was 0.78 (95% confidence interval: 0.52–1.16, I^2^ = 0%; Fig. 1) [18, 25]. We refrained from pooling results from the two studies that assessed the association between MetS and subclinical hypothyroidism as the results were deemed to be very heterogeneous (I^2^ = 92.3%) [18, 25]. We found only one study that reported the association between MetS and hyperthyroidism [25]. The unadjusted ORs for the association between MetS and overt and subclinical hyperthyroidism were 1.15 (0.63–2.09) and 1.69 (1.02–2.79), respectively [25].

### Diabetes mellitus

**Fig. 2:**
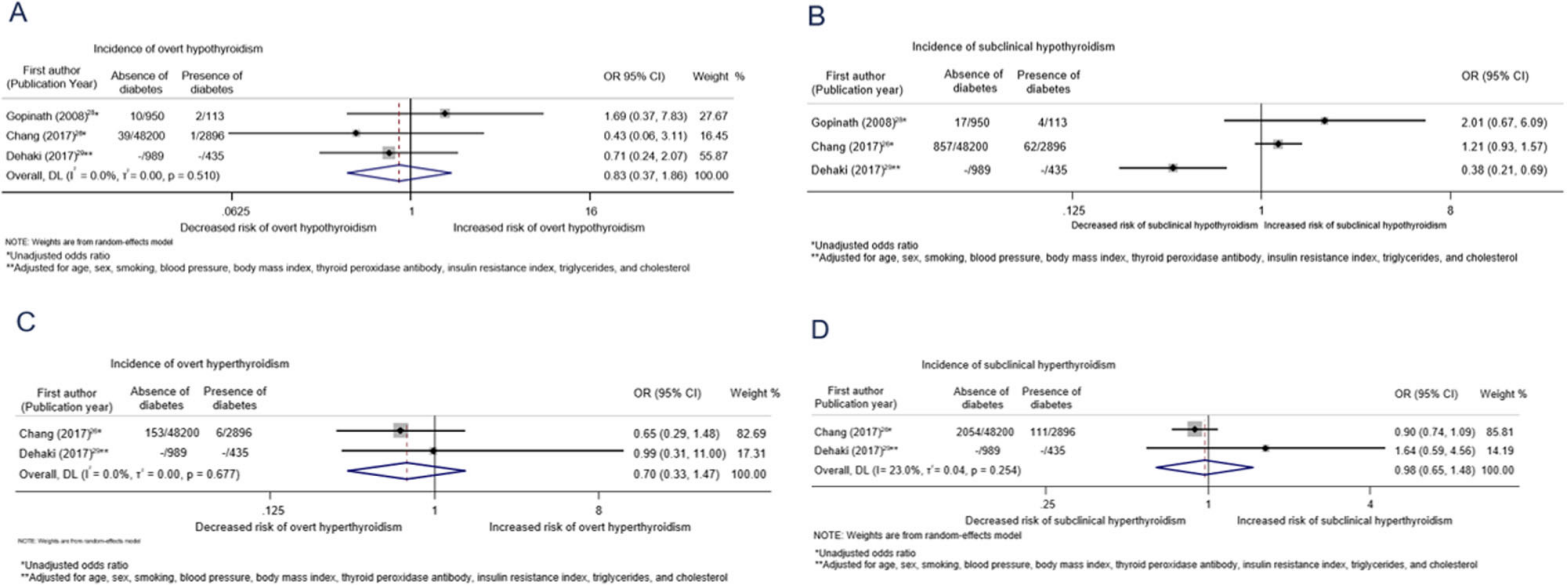
Association between diabetes mellitus at baseline and thyroid disorders at follow-up. **A** Association between diabetes mellitus and incidence of overt hypothyroidism. **B** Association between diabetes mellitus and incidence of subclinical hypothyroidism. **C** Association between diabetes mellitus and incidence of overt hyperthyroidism. **D** Association between diabetes mellitus and incidence of subclinical hyperthyroidism

The pooled unadjusted OR from three studies for the association between diabetes mellitus and incidence of overt hypothyroidism was 0.83 (0.37–1.86, I^2^ = 0%; Fig. 2). We were unable to pool results for the association between diabetes and subclinical hypothyroidism (I^2^ = 85.3%) [30, 32, 33]. Similar results were found for the association between diabetes mellitus and overt and subclinical hyperthyroidism after pooling unadjusted ORs from two studies [30, 33].

### Prediabetes

**Fig. 3:**
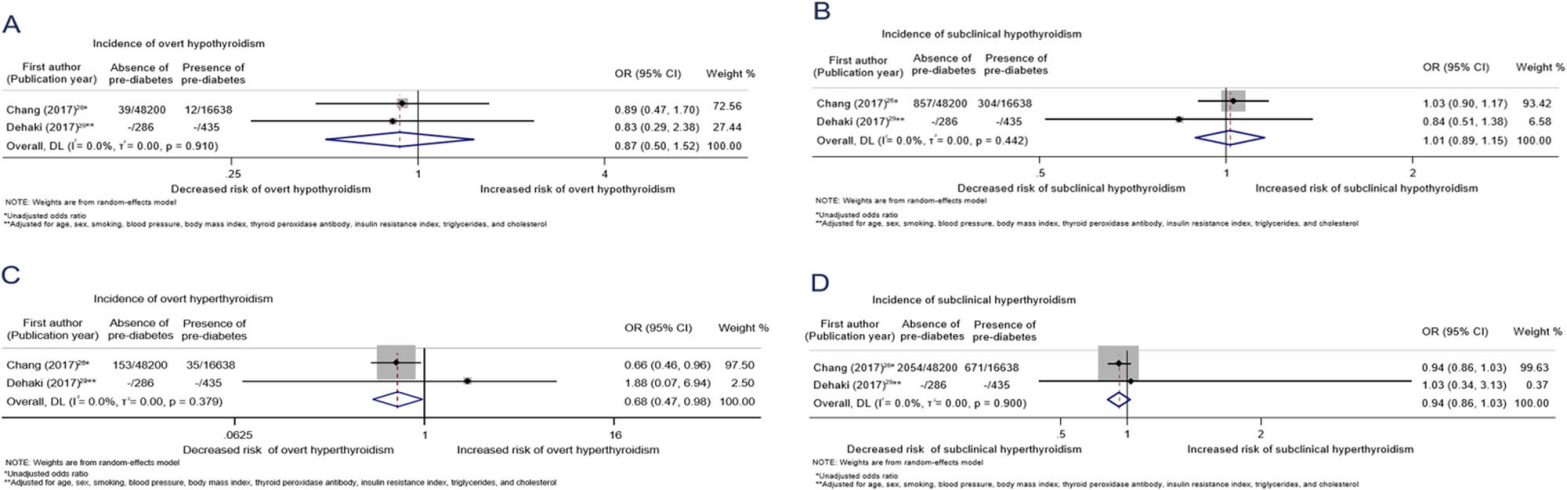
Association between prediabetes at baseline and thyroid disorders at follow-up. **A** Association between prediabetes and incidence of overt hypothyroidism. **B** Association between prediabetes and incidence of subclinical hypothyroidism. **C** Association between prediabetes and incidence of overt hyperthyroidism. **D** Association between prediabetes and incidence of subclinical hyperthyroidism

After pooling results from two studies [30, 33], the overall OR was 0.87 (0.50–1.52, I^2^ = 0%) and 1.01 (0.89–1.15, I^2^ = 0%) for the association between prediabetes and overt and subclinical hypothyroidism, respectively (Fig. 3). Pre-diabetes was defined as a fasting glucose between 5.6 and 6.9 mmol/l (or additionally as a two-hour blood glucose between 7.8 and 11.0 mmol/l in one study) [30, 33]. There was a lower odds of developing overt hyperthyroidism in individuals with prediabetes at baseline (pooled OR: 0.68 (0.47–0.98), I^2^ = 0%) [30, 33], but the evidence came predominantly from one study [30], which received 97.5% of the weight in the meta-analysis and the effect estimate was not adjusted for possible confounders. The OR for the association between prediabetes and subclinical hyperthyroidism was 0.94 (0.86–1.03, I^2^ = 0) [30, 33].

### Obesity

**Fig. 4:**
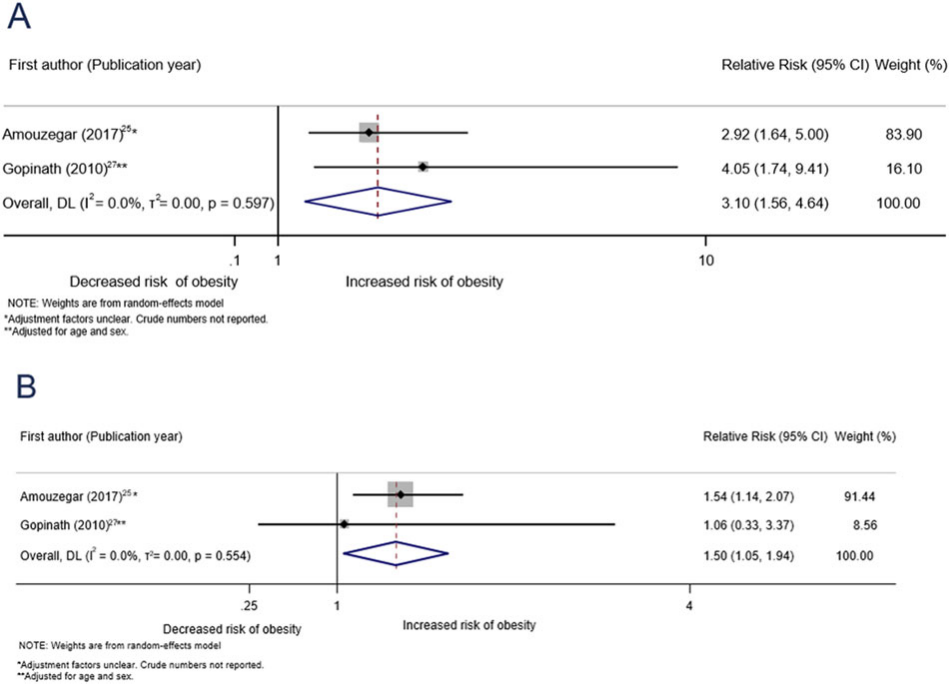
Association between obesity (body mass index ≥30 kg/m^2^) at baseline and hypothyroidism at follow-up. **A** Association between obesity and incidence of overt hypothyroidism. **B** Association between obesity and incidence of subclinical hypothyroidism

Only one study evaluated the association between abdominal obesity and subclinical hypothyroidism (adjusted HR: 1.07 (0.93–1.25)) and overt hypothyroidism (unadjusted OR: 1.32 (0.72–2.42); adjusted HR unavailable) [18]. Two studies assessed the association between obesity (defined as BMI ≥ 30k kg/m^2^) and overt and subclinical hypothyroidism [29, 31]. Both studies found evidence of an association between obesity at baseline and incidence of overt hypothyroidism (pooled RR: 3.10 (1.56–4.64), I^2^ = 0%; Fig. 4)) [29, 31]. We also found evidence of an increased risk of subclinical hypothyroidism in individuals with obesity at baseline (pooled RR 1.50 (1.05–1.94) for two studies, I^2^ = 0%)) [29, 31]. We identified one study that investigated the association between obesity and Grave’s hyperthyroidism among women [34]. Grave’s hyperthyroidism is an autoimmune form of hyperthyroidism and the outcome was self-reported in this study [34]. This study found that obesity was associated with a decreased risk of Grave’s hyperthyroidism (hazard ratio: 0.68 (0.49–0.92)). We did not identify any studies assessing the association between obesity and subclinical hyperthyroidism.

### Hypertension

Only one study reported the association between hypertension and overt hypothyroidism (RR: 1.68 (1.53–1.84)) [29]. Similarly, only one study reported the result of the association between hypertension and subclinical hypothyroidism (adjusted HR: 1.24 (1.04–1.48)) [18]. No studies were found on the association between hypertension at baseline and incidence of overt or subclinical hyperthyroidism.

### Dyslipidemia

We were unable to pool results for the association between dyslipidemia and any of the outcomes due to the heterogeneity of the results and/or lack of data. One study found evidence of an association between high triglycerides and overt hypothyroidism (RR: 1.79, (1.15–2.79)) as well as high total cholesterol and subclinical hypothyroidism (RR: 1.60 (1.15–2.23)) [29]. Another study also reported an increased risk of subclinical hypothyroidism in individuals with hypertriglyceridemia (adjusted HR: 1.18 (1.00–1.39)) [18].

## Discussion

The aim of this systematic review and meta-analysis was to summarize the available evidence on the association between MetS, and its components, and incidence of thyroid diseases. We found very few studies that assessed these associations and it was not always possible to pool effect estimates due to large heterogeneity across the studies or due to different effect measures reported. Overall, we found no clear evidence on the association between MetS at baseline and incidence of overt or subclinical hypothyroidism. However, we did find an association between obesity and both overt and subclinical hypothyroidism.

There is a large body of literature on the association between MetS and thyroid diseases, the vast majority of which originates from cross-sectional or longitudinal studies that hypothesized that thyroid diseases increase the risk of MetS [11–13, 19–21]. Conversely, there are only a few studies that have assessed the inverse association, i.e., whether MetS at baseline increases the risk of developing thyroid disease. We were able to include only two studies in our meta-analysis on the association between MetS and overt hypothyroidism and we were unable to draw any firm conclusions on the nature of the association. Similarly, the heterogeneity of results from primary studies hampered our ability to reach a definite conclusion on the association between MetS and hyperthyroidism. An individual participant data analysis may help elucidate these associations, as it will allow for the standardization of definitions and statistical methods.

In theory, it is plausible that MetS and its components can influence thyroid hormones. For example, although obesity is usually considered a result of hypothyroidism, recent studies suggest that obesity can play a causal role in the development of thyroid diseases [25, 35]. Inflammatory markers such as cytokines and interleukins are elevated in individuals who are obese [25, 35]. These inflammatory markers in turn may inhibit the mRNA expression of symporter sodium/iodide, which then affects the uptake of iodine into thyroid cells [25, 35]. Leptin can also play a role by inducing morphological changes in the thyroid gland and reducing the expression of sodium/iodide symporter and thyroglobulin [35]. Moreover, it has been shown that cytokines can have an inhibitory role on deiodinases [25, 36]. In line with these results, we found two studies showing evidence of a positive association between obesity at baseline and incidence of overt hypothyroidism [29, 31]. A previous systematic review and meta-analysis found an association between obesity and overt hypothyroidism (RR 3.21 (2.12–4.86)), although most of the included studies were cross-sectional [35] while we only included prospective cohorts. In one cross-sectional study, the authors investigated the use of nationwide data from Korea, to determine whether there is a correlation between thyroid hormones and metabolically healthy and unhealthy obese and non-obese subjects [37]. It was found that decreased TSH and increased FT4 levels, within the reference range, were associated with metabolically healthy non-obesity and that the association was modified by gender and age [37].

Diabetes and thyroid diseases are common endocrine disorders and they tend to coexist [7]. Although it is widely accepted that there is an association between type 1 diabetes and thyroid dysfunction, and consequently guidelines recommend screening individuals with type 1 diabetes for thyroid disorders [38], the association is less evident for type 2 diabetes. Results from cross-sectional studies and longitudinal studies assessing the risk of diabetes among individuals with thyroid disorders have been inconclusive [12, 19, 20, 22, 39–41]. However, very few studies have assessed the association in the inverse direction, i.e., by examining whether individuals with type 2 diabetes are at an increased risk of developing thyroid disease. We were able to include only two studies in our meta-analysis on the association between diabetes and hypothyroidism and results were inconclusive. Findings were similar for prediabetes, with the exception of a decreased risk of overt hyperthyroidism among individuals with prediabetes. Several mechanisms have been proposed to explain how glucose metabolism influences the development of thyroid disorders. One hypothesis is that hyperglycemia can affect TSH secretion from the hypothalamus [7]. It is also believed that hyperglycemia has an effect on the TSH response to thyrotropin-releasing hormone and can influence the conversion of free thyroxine to free triiodothyronine in peripheral tissues [7].

We were unable to pool results for the association between hypertension and hypothyroidism as we only found one study that examined overt hypothyroidism [29] and another that examined subclinical hypothyroidism [18]. Both studies reported an increased risk of hypothyroidism (overt or subclinical) among hypertensive individuals at baseline [18, 29]. Although it was not possible to perform a quantitative synthesis, we found two studies reporting evidence of a positive association between dyslipidemia and hypothyroidism. It has been shown in animal models that a high-fat diet and excess iodine can lead to thyroid structural changes and disorders in thyroid hormones [42]. A recent study performing a 2-sample bidirectional Mendelian randomization (MR) analysis, using summary statistics from large-scale genome-wide association studies of TSH, FT4, and blood lipids, suggested that even within the reference range, higher TSH or lower FT4 are causally associated with increased total cholesterol and low-density lipoprotein, although no reverse causal association was detected [43].

Gaps in our current mechanistic knowledge concerning the reported associations may limit the ability to carry out accurate diagnosis and effective treatment, as, for instance, the role of tissue deiodinases is usually not considered. In this context, liver dysfunction of type 3 deiodinase (D3) may result in decreased production of T3, which contributes to metabolic-associated fatty liver disease (MAFLD) [44].

The main limitations of this analysis are related to the use of aggregate-level data. Only a few studies met our inclusion criteria and it was not always possible to perform a meta-analysis due to the large heterogeneity we found in results from primary studies. Moreover, most of the estimates lacked adjustment for confounding factors as odds ratios were calculated from raw outcome data, which reduced the quality of the included estimates and correspondingly increased the risk of bias. Furthermore, we elected to pool together relative risks and odds ratios from studies when event rates are low. However, concerning the association between obesity and hypothyroidism, the event rate for overt hypothyroidism was 6.2%. Our study also has several strengths. We only included prospective cohorts. The comprehensive nature of this systematic review, overseen by medical librarians, reduces the possibility of missing relevant studies. Moreover, we adhered to a predefined protocol and two authors were involved in study selection and study quality judgement to maximize reproducibility.

## Conclusions

In this systematic review and meta-analysis, we were unable to draw firm conclusions regarding the association between MetS and its components and thyroid disease owing to the limited number of studies and the heterogeneous reporting of results. However, an association between obesity at baseline and incidence of hypothyroidism was observed. Additional larger studies as well as individual participant data meta-analyses that standardize definitions and statistical methods are warranted to help elucidate these associations.

### Supplementary Information

Supplementary_files_13.05.23
