# Supplementary material for: A systematic review and meta-analysis investigating the relationship between metabolic syndrome and the incidence of thyroid diseases
Source: Endocrine. 2023 Sep 9;84(2):320–7. doi: 10.1007/s12020-023-03503-7 (PMC11076217; doi:10.1007/s12020-023-03503-7)
Supplement: Supplementary file 1 — Supplementary_files_13.05.23 [file 12020_2023_3503_MOESM1_ESM.docx]

**Supplementary Information**

**Article title:** A systematic review and meta-analysis of the association between metabolic syndrome and the incidence of thyroid diseases: an attempt to unravel a chicken and egg dilemma

**Journal:** Endocrine

**Authors**: Heba Alwan, Valerie Aponte Ribero, Orestis Efthimiou, Cinzia Del Giovane, Nicolas Rodondi, Leonidas Duntas

**Corresponding author:**

Heba Alwan

Institute of Primary Health Care (BIHAM)

University of Bern

Mittelstrasse 43, 3012 Bern, Switzerland

Phone: 00 41 31 684 57 93

Email: heba.al-alwan@biham.unibe.ch

**Supplementary Figure 1.** Flow diagram of the systematic review and study selection

Records identified through database searching

N = 4061

Records after duplicates removed

N = 2927

Duplicates Removed
(using Deduklick*)

N = 1134

Full text screening

N = 17

Excluded based on title and abstract

N = 2910

Full text articles included in qualitative synthesis

N = 8

Excluded based on full-text screening

N = 9

- 1 was not a longitudinal design

- 2 did not include any exposures of interest

- 1 did not have a control group

- 2 did not report the outcomes of interest

- 2 did not report results separately for overt and subclinical thyroid disease

- 1 used the same source population as another article and did not report sufficient data

Full text articles included in quantitative synthesis

N = 7

*doi.org/10.1186/s13643-022-02045-9

**Supplementary Table 1. Newcastle Ottawa Scale of studies included in the quantitative analysis**

The first category refers to the representativeness of the study. The criterion was met if conducted in the general population, and representative of the source population. The second category refers to selection of the non-exposed. Criterion was met if studies had controls. The third category refers to ascertainment of exposure. The criterion was met if studies used an appropriate exposure measurement (e.g. serum measurement of glucose, triglycerides, etc). The fourth category refers to demonstration that outcome of interest was not present at start of the study. Criterion was met if individuals with thyroid dysfunction were excluded from analyses. The fifth category refers to comparability on the basis of the design or analysis. Criterion was met when analyses were adjusted for at least age. The sixth category refers to assessment of the outcome, criterion was met if data on thyroid status at follow-up was available. The seventh category refers to the length of follow-up. Criterion was met if the follow-up was at least one year. The category eight refers on the adequacy of follow-up, criterion was met if the loss of follow up was ≤ 20%. We considered the final NOS quality score of studies for judging the study limitations (risk of bias) in the GRADE assessment

| Study | Selection | | | | Comparability | Outcome | | |  |
| --- | --- | --- | --- | --- | --- | --- | --- | --- | --- |
|  | **Representativeness of exposed cohort** | **Selection of the non exposed cohort** | **Ascertainment of exposure** | **Demonstration that outcome of interest was not present at start of the study** | **Comparability of cohorts on the basis of the design or analysis** | **Assessment of outcome** | **Was follow-up long enough for outcomes to occur** | **Adequacy of follow up (Loss of follow-up)** | **Quality Score** |
| Amouzegar 2017 (25) | ★ | ★ | ★ | ★ | - | ★ | ★ | ★ | 7 |
| Chang 2017 (14) | **-** | ★ | ★ | ★ | - | ★ | ★ | ★ | 6 |
| Chang 2017 (26) | **-** | ★ | ★ | ★ | - | ★ | ★ | ★ | 6 |
| Dehaki 2017 (29) | ★ | ★ | ★ | ★ | ★★ | ★ | ★ | ★ | 9 |
| Gopinath 2008 (28) | ★ | ★ | ★ | ★ | - | ★ | ★ | - | 6 |
| Gopinath 2010 (27) | ★ | ★ | ★ | ★ | ★★ | ★ | ★ | - | 8 |
| Mehran 2020 (21) | ★ | ★ | ★ | ★ | - | ★ | ★ | ★ | 7 |

Thresholds for converting the Newcastle-Ottawa scales to AHRQ standards (good, fair, and poor): Good quality: 3 or 4 stars in selection domain AND 1 or 2 stars in comparability domain AND 2 or 3 stars in outcome/exposure domain Fair quality: 2 stars in selection domain AND 1 or 2 stars in comparability domain AND 2 or 3 stars in outcome/exposure domain Poor quality: 0 or 1 star in selection domain OR 0 stars in comparability domain OR 0 or 1 stars in outcome/exposure domain.

**Appendix. Search strategy for Ovid Medline, Embase.com, and Cochrane CENTRAL (February 22, 2023)**

**Ovid Medline ALL**

(((obesity/ OR abdominal obesity/ OR exp abdominal fat/ OR waist circumference/ OR waist-hip ratio/ OR (obes* OR adipos* OR ((abdom* OR body OR intraabdominal OR visceral) ADJ3 fat) OR (waist ADJ3 (circumference* OR hip))).ab,ti.) OR (exp hypertension/ OR (hypertensi* OR ((high OR increased OR elevated OR raised) ADJ3 blood pressure*)).ab,ti.) OR (glucose intolerance/ OR glucose tolerance test/ OR blood glucose/ OR insulin resistance/ OR insulin/bl OR hyperglycemia/ OR hypoglycemia/ OR hyperinsulinism/ OR metabolic syndrome/ OR cardiometabolic risk factors/ OR glycated hemoglobin/ OR (((glucose OR sugar OR insulin*) ADJ3 (resistan* OR level* OR blood OR serum OR plasma* OR concentration* OR tolerance OR intolerance OR sensitiv* OR insensitiv* OR response OR dependen* OR homeosta* OR load*)) OR ((fasting OR impaired OR high OR elevated) ADJ2 (glucose OR insulin OR proinsulin OR sugar OR glycemi* OR glycaemi*)) OR hyperglycemi* OR hyperglycaemi* OR dysglycemi* OR dysglycaemi* OR metabolic syndrom* OR syndrom* X OR glycated hemoglobin* OR glycated haemoglobin* OR glycosylated haemoglobin* OR glycosylated hemoglobin* OR HbA1c OR Hb A1c OR hemoglobin A1c OR haemoglobin A1c OR HOMA-IR).ab,ti.) OR (exp diabetes mellitus, type 2/ OR latent autoimmune diabetes in adults/ OR (((diabet* OR dm) ADJ3 (type 2 OR type2 OR type ii OR non insulin OR noninsulin OR insulin independent OR adult onset OR slow onset OR maturity onset OR ketosis resistant OR latent OR late onset)) OR prediabet* OR pre-diabet* OR praediabet* OR T2DM OR dmt2 OR dm2 OR T2-DM OR dm-t2 OR dm-2 OR NIDDM OR NID-DM).ab,ti.) OR (exp dyslipidemias/ OR exp hypertriglyceridemia/ OR lipid blood level/ OR cholesterol, HDL/bl OR triglycerides/bl OR (dyslipidemi* OR dyslipidaemi* OR lipidemi* OR lipidaemi* OR hyperlipidemi* OR hyperlipidaemi* OR hypertriglyceridemi* OR hypertriglyceridaemi* OR triglyceridemi* OR triglyceridaemi* OR hyperlipoproteinemi* OR hyperlipoproteinaemi* OR hypercholesterolemi* OR hypercholesterolaemi* OR ((cholester* OR HDL OR lipoprotein* OR triacylglycerol* OR triglyceride*) ADJ6 (level* OR blood OR serum OR plasma* OR concentration* OR profile*))).ab,ti.)) AND (*thyroid function tests/ OR exp *thyroid diseases/ OR *thyrotropin/ OR *thyroid hormones/ OR *thyroxine/ OR *triiodothyronine/ OR ((thyroid* ADJ3 (function* OR dysfunction* OR hypofunction* OR disorder* OR disease* OR autoimmun* OR auto-immun* OR hormone* OR autoantibod* OR antibod*)) OR thyroidit* OR hyperthyro* OR hypothyro* OR thyrotropin* OR thyroid-stimulating hormone* OR ((t4 OR ft4 OR t-4 OR ft-4 OR tsh OR liothyronin* OR thyroxin*) ADJ3 (free OR plasma OR blood OR serum OR level* OR concentrat* OR low OR high OR elevat* OR decrease* OR increase*)) OR (thyroid* ADJ3 peroxidase* ADJ3 antibod*) OR ((tpo OR thyroglobulin* OR thyroperoxidas* OR thyroperoxid*) ADJ3 (antibod* OR positiv* OR negativ* OR status*)) OR triiodothyronin* OR triodothyronin* OR liothyronin* OR thyroxin* OR TPOAb OR euthyroid* OR graves OR goiter).ti,kf) AND (cohort studies/ OR longitudinal studies/ OR prospective studies/ OR follow-up studies/ OR (cohort* OR (observational* ADJ3 stud*) OR prospectiv* OR longitudinal* OR follow up OR followed up OR followup OR (compar* ADJ10 group*)).ab,ti.) AND (prevalence/ OR incidence/ OR epidemiology.fs. OR (risk* OR prevalen* OR incidence* OR predict* OR associat*).ab,ti.)) NOT (exp animals/ NOT humans/) NOT (letter OR news OR comment OR editorial OR congress).pt. NOT ("systematic review"/ OR meta-analysis/ OR systematic review.ti. OR meta-analysis.ti.) NOT (case reports/ OR case-report.ti.) NOT ((exp infant/ or exp child/ or adolescent/) NOT (exp adult/))

**Embase.com** (via https://www.embase.com)

(((obesity/de OR 'abdominal obesity'/de OR 'abdominal fat'/exp OR 'diabetic obesity'/de OR 'body mass'/de OR 'waist circumference'/de OR 'waist hip ratio'/de OR (obes* OR adipos* OR ((abdom* OR body OR intraabdominal OR visceral) NEAR/3 fat) OR (waist NEAR/3 (circumference* OR hip))):ab,ti) OR ('hypertension'/exp OR 'elevated blood pressure'/de OR (hypertensi* OR ((high OR increased OR elevated OR raised) NEAR/3 'blood pressure*')):ab,ti) OR ('impaired glucose tolerance'/exp OR 'glucose tolerance test'/exp OR 'glucose intolerance'/exp OR 'impaired fasting glucose'/de OR 'fasting glucose'/de OR 'insulin response'/exp OR 'glucose blood level'/exp OR 'insulin blood level'/exp OR 'hyperglycemia'/de OR 'dysglycemia'/de OR 'metabolic syndrome X'/exp OR 'cardiometabolic risk factor'/de OR 'glycosylated hemoglobin'/exp OR (((glucose OR sugar OR insulin*) NEAR/3 (resistan* OR level* OR blood OR serum OR plasma* OR concentration* OR tolerance OR intolerance OR sensitiv* OR insensitiv* OR response OR dependen* OR homeosta* OR load*)) OR ((fasting OR impaired OR high OR elevated) NEAR/2 (glucose OR insulin OR proinsulin OR sugar OR glycemi* OR glycaemi*)) OR hyperglycemi* OR hyperglycaemi* OR dysglycemi* OR dysglycaemi* OR 'metabolic syndrom*' OR 'syndrom* X' OR 'glycated hemoglobin*' OR 'glycated haemoglobin*' OR 'glycosylated haemoglobin*' OR 'glycosylated hemoglobin*' OR HbA1c OR 'Hb A1c' OR 'hemoglobin A1c' OR 'haemoglobin A1c' OR 'HOMA-IR'):ab,ti) OR ('non insulin dependent diabetes mellitus'/de OR 'latent autoimmune diabetes in adults'/exp OR (((diabet* OR dm) NEAR/3 ('type 2' OR type2 OR 'type ii' OR 'non insulin' OR noninsulin OR 'insulin independent' OR 'adult onset' OR 'slow onset' OR 'maturity onset' OR 'ketosis resistant' OR latent OR 'late onset')) OR prediabet* OR 'pre-diabet*' OR praediabet* OR T2DM OR dmt2 OR dm2 OR T2-DM OR dm-t2 OR dm-2 OR NIDDM OR 'NID-DM'):ab,ti) OR ('dyslipidemia'/de OR 'hypertriglyceridemia'/exp OR 'lipid blood level'/exp OR (dyslipidemi* OR lipidemi* OR lipidaemi* OR dyslipidaemi* OR hyperlipidemi* OR hyperlipidaemi* OR hypertriglyceridemi* OR hypertriglyceridaemi* OR triglyceridemi* OR triglyceridaemi* OR hyperlipoproteinemi* OR hyperlipoproteinaemi* OR hypercholesterolemi* OR hypercholesterolaemi* OR ((cholester* OR HDL OR lipoprotein* OR triacylglycerol* OR triglyceride*) NEAR/6 (level* OR blood OR serum OR plasma* OR concentration* OR profile*))):ab,ti)) AND ('thyroid function'/mj OR 'thyroid function test'/mj OR 'thyroid disease'/exp/mj OR 'thyrotropin'/mj OR 'thyrotropin blood level'/mj OR 'thyroid hormone'/mj OR 'thyromimetic agent'/exp/mj OR 'thyroid hormone blood level'/exp/mj OR 'thyroid peroxidase antibody'/exp/mj OR 'thyroglobulin antibody'/mj OR 'thyroxine'/mj OR 'thyrotropin'/mj OR ((thyroid* NEAR/3 (function* OR dysfunction* OR hypofunction* OR disorder* OR disease* OR autoimmun* OR auto-immun* OR hormone* OR autoantibod* OR antibod*)) OR thyroidit* OR hyperthyro* OR hypothyro* OR thyrotropin* OR 'thyroid-stimulating hormone*' OR ((t4 OR ft4 OR t-4 OR ft-4 OR tsh OR liothyronin* OR thyroxin*) NEAR/3 (free OR plasma OR blood OR serum OR level* OR concentrat* OR low OR high OR elevat* OR decrease* OR increase*)) OR (thyroid* NEAR/3 peroxidase* NEAR/3 antibod*) OR ((tpo OR thyroglobulin* OR thyroperoxidas* OR thyroperoxid*) NEAR/3 (antibod* OR positiv* OR negativ* OR status*)) OR triiodothyronin* OR triodothyronin* OR liothyronin* OR thyroxin* OR TPOAb OR euthyroid* OR graves OR goiter):ti,kw) AND ('cohort analysis'/exp OR 'longitudinal study'/exp OR 'prospective study'/exp OR 'follow up'/exp OR (cohort* OR (observational* NEAR/3 stud*) OR prospectiv* OR longitudinal* OR 'follow up' OR 'followed up' OR followup OR (compar* NEAR/10 group*)):ab,ti) AND ('prevalence'/de OR 'incidence'/de OR 'epidemiological data'/de OR (risk* or prevalen* or incidence* or predict* or associat*):ab,ti)) NOT ([animals]/lim NOT [humans]/lim) NOT ([Conference Abstract]/lim OR [Letter]/lim OR [Note]/lim OR [Editorial]/lim OR [Review]/lim OR [Preprint]/lim) NOT ('systematic review'/de OR 'meta analysis'/de) NOT ('case report'/de OR case-report:ti) NOT ('randomized controlled trial'/exp NOT 'cohort analysis'/exp) NOT (([infant]/lim OR [child]/lim OR [adolescent]/lim) NOT [adult]/lim)

**Cochrane Central Register of Controlled Trials** (via Wiley)

#1 (obes* OR adipos* OR ((abdom* OR body OR intraabdominal OR visceral) NEAR/3 fat) OR (waist NEAR/3 (circumference* OR hip))):ab,ti 59544

#2 (hypertensi* OR ((high OR increased OR elevated OR raised) NEAR/3 "blood pressure")):ab,ti 66279

#3 (((glucose OR sugar OR insulin*) NEAR/3 (resistan* OR level* OR blood OR serum OR plasma* OR concentration* OR tolerance OR intolerance OR sensitiv* OR insensitiv* OR response OR dependen* OR homeosta* OR load*)) OR ((fasting OR impaired OR high OR elevated) NEAR/2 (glucose OR insulin OR proinsulin OR sugar OR glycemi* OR glycaemi*)) OR hyperglycemi* OR hyperglycaemi* OR dysglycemi* OR dysglycaemi* OR (metabolic NEXT syndrom*) OR (glycated NEXT hemoglobin*) OR (glycated NEXT haemoglobin*) OR (glycosylated NEXT haemoglobin*) OR (glycosylated NEXT hemoglobin*) OR HbA1c OR "Hb A1c" OR "hemoglobin A1c" OR "haemoglobin A1c" OR "HOMA-IR"):ab,ti 82604

#4 (((diabet* OR dm) NEAR/3 ("type 2" OR type2 OR "type ii" OR "non insulin" OR noninsulin OR "insulin independent" OR "adult onset" OR "slow onset" OR "maturity onset" OR "ketosis resistant" OR latent OR "late onset")) OR prediabet* OR (pre NEXT diabet*) OR praediabet* OR T2DM OR dmt2 OR dm2 OR "T2-DM" OR "dm-t2" OR "dm-2" OR NIDDM OR "NID-DM"):ab,ti 48869

#5 (dyslipidemi* OR dyslipidaemi* OR lipidemi* OR lipidaemi* OR hyperlipidemi* OR hyperlipidaemi* OR hypertriglyceridemi* OR hypertriglyceridaemi* OR triglyceridemi* OR triglyceridaemi* OR hyperlipoproteinemi* OR hyperlipoproteinaemi* OR hypercholesterolemi* OR hypercholesterolaemi* OR ((cholester* OR HDL OR lipoprotein* OR triacylglycerol* OR triglyceride*) NEAR/6 (level* OR blood OR serum OR plasma* OR concentration* OR profile*))):ab,ti 40142

#6 #1 OR #2 OR #3 OR #4 OR #5 211548

#7 ((thyroid* NEAR/3 (function* OR dysfunction* OR hypofunction* OR disorder* OR disease* OR autoimmun* OR "auto-immune" OR hormone* OR autoantibod* OR antibod*)) OR thyroidit* OR hyperthyro* OR hypothyro* OR thyrotropin* OR ("thyroid-stimulating" NEXT hormone*) OR ((t4 OR ft4 OR tsh OR liothyronin* OR thyroxin*) NEAR/3 (free OR plasma OR blood OR serum OR level* OR concentrat* OR low OR high OR elevat* OR decrease* OR increase*)) OR (thyroid* NEAR/3 peroxidase* NEAR/3 antibod*) OR ((tpo OR thyroglobulin* OR thyroperoxidas* OR thyroperoxid*) NEAR/3 (antibod* OR positiv* OR negativ* OR status*)) OR triiodothyronin* OR triodothyronin* OR liothyronin* OR thyroxin* OR TPOAb OR euthyroid* OR graves OR goiter):ti,kw 6954

#8 #6 AND #7 1015

#9 (cohort* OR (observational* NEAR/3 stud*) OR prospectiv* OR longitudinal* OR "follow up" OR "followed up" OR followup OR (compar* NEAR/10 group*)):ab,ti 627912

#10 #8 AND #9 401

#11 (risk* OR prevalen* OR incidence* OR predict* OR associat*):ab,ti 635016

#12 #10 AND #11 223 (9 Cochrane Reviews, 214 Trials)
